# *Sulfolobus acidocaldarius*, half a century of archaeal research

**DOI:** 10.1128/jb.00322-26

**Published:** 2026-08-06

**Authors:** Marleen van Wolferen, Michael Spädt, Shamphavi Sivabalasarma, Sonja-Verena Albers

**Affiliations:** 1 Faculty of Biology, Molecular Biology of Archaea, University of Freiburg 98898, Freiburg, Germany; 2 CIBSS-Centre for Integrative Biological Signaling Studies, Freiburg, Germany; University of Florida, Gainesville, Florida, USA

**Keywords:** Archaea, model organism, *Sulfolobus acidocaldarius*, cell biology, molecular biology, history, genetics, biochemistry, S-layer, T4P

## Abstract

Since its isolation from a Yellowstone hot spring in 1972, *Sulfolobus acidocaldarius* has become one of the most important model organisms for archaeal biology. Initially studied for its remarkable adaptation to high temperature and low pH, it has evolved into a genetically tractable system that has contributed substantially to our understanding of archaeal physiology, molecular biology, and evolution. Here, we summarize more than 5 decades of research on *S. acidocaldarius,* highlighting key developments in genetic and microscopy tools and their impact on our understanding of archaeal cell biology. Studies in this organism have improved our understanding of archaeal metabolism, chromosome organization, DNA replication and segregation, cell division, protein glycosylation, biofilm formation, and the assembly and regulation of archaeal surface structures, including archaella and type IV pili. Beyond fundamental biology, *S. acidocaldarius* has also served as a valuable source of thermostable enzymes and other biomolecules with biotechnological potential. Today, *S. acidocaldarius* is still one of the main model organisms for archaeal biology. Ongoing advances in genetics, imaging, and structural biology continue to expand its experimental potential, while its phylogenetic position within the Thermoproteota makes it a powerful system for investigating the evolutionary origins of eukaryotic cellular complexity.

## INTRODUCTION

Research on the molecular biology of Archaea has long lagged behind studies of bacteria and eukaryotes. This gap was due, in part, to the initial absence of genetic tools and a general lack of interest in archaeal organisms within the wider microbial community. Unlike bacteria, archaea include no known pathogens, which limited both clinical relevance and funding opportunities. For many years, Archaea were viewed as oddities: extremophiles surviving in harsh environments, but not central to mainstream biology. That perception began to change in the 1970s and early 1980s. Early biochemical studies by researchers including Otto Kandler revealed fundamental differences between archaeal and bacterial cell envelopes, such as the absence of classical peptidoglycan and the presence of unique cell wall polymers in methanogens (1). Shortly thereafter, the pioneering work of Carl Woese and George Fox, based on 16S rRNA phylogenies, demonstrated that archaea constitute a separate domain of life (2). In 1990, Woese, Kandler, and Mark Wheelis formally proposed the three-domain tree of life (3).

Around the same time, researchers including Wolfram Zillig and Karl Stetter characterized the unusual molecular biology of thermophilic archaea, including unique RNA polymerases and transcription systems (4–6). Among early archaeal isolates, members of the genus *Sulfolobus* played a crucial role in defining the archaeal domain. These organisms combined bacterial-like metabolic traits with eukaryotic-style features in their information-processing systems, highlighting the evolutionary bridge that Archaea represent between the other two domains of life.

Despite early skepticism about extremophiles as model organisms, *Sulfolobus* gained attention as genome sequencing and molecular tools improved. As researchers came to appreciate the fundamental differences between archaeal and bacterial systems, including differences in transcription machinery, membrane lipids, and cell walls, it became increasingly clear that dedicated archaeal models were essential. However, the unique biology of Archaea also complicated genetic work, as the targets of common bacterial selection markers (e.g., antibiotics) often differed significantly and were ineffective in archaeal cells. In addition, these compounds were often not stable under the harsh growth conditions of many archaeal model organisms (7).

## ARCHAEA GENETIC SYSTEMS

The first breakthroughs in archaeal genetics came in the 1990s and early 2000s in the phylum Euryarchaeota. Methanogens such as *Methanosarcina acetivorans* and *Methanococcus maripaludis* (8, 9), as well as halophiles like *Haloferax volcanii* (10–14), became tractable model systems. These organisms were genetically accessible through auxotrophic selection and plasmid-based tools. Later, thermophilic euryarchaea such as *Thermococcus kodakarensis* and *Pyrococcus furiosus* became genetically modifiable (15, 16). More recently, advances were made in other methanogens, including *Methanothermobacter thermautotrophicus* (17, 18), expanding the molecular toolbox within Euryarchaeota. In contrast, other archaeal phyla remained genetically inaccessible for a long time. This was especially true for the Thermoproteota (formerly Crenarchaeota), despite their central importance for understanding archaeal cell biology and evolution.

Compared with Euryarchaeota, members of the Thermoproteota have fundamentally distinct cellular biology, metabolic strategies, and ecological adaptations, underscoring the need for robust genetic systems to study them. Moreover, they played an important role in early views on archaeal evolution. As early as the 1980s, Wolfram Zillig argued that many “eukaryotic” molecular features, particularly in transcription and replication, were best explained by an origin of eukaryotes from within the Archaea, with sulfur-dependent lineages such as *Sulfolobus* playing a key role in this ancestry. His idea anticipated the now widely accepted view that eukaryotes emerged from within the archaeal domain (19). This perspective gained strong support with the discovery of the Asgard archaea (20, 21), now recognized as the closest archaeal relatives of eukaryotes. Phylogenetic analyses place Thermoproteota closer to Asgard archaea than Euryarchaeota are to the Asgard archaea. Consistent with this relationship, Thermoproteota share several notable features with Asgard lineages, including ESCRT-III-based membrane remodeling systems (discussed below). However, Asgard archaea currently remain genetically intractable, making genetically accessible Thermoproteota particularly powerful model systems for addressing evolutionary questions that cannot yet be addressed directly in Asgard lineages.

## ISOLATION AND TAXONOMY OF SULFOLOBALES

**Fig 1:**
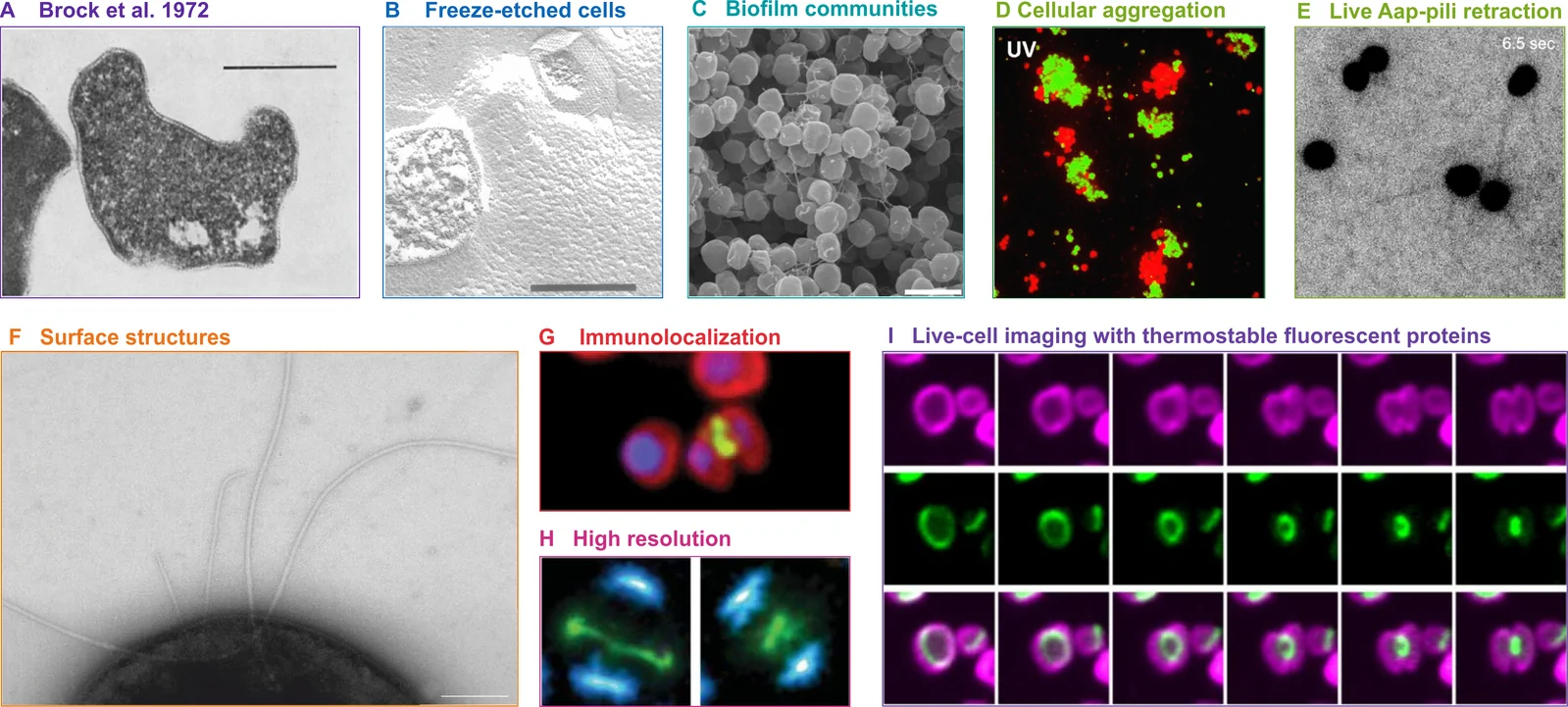
Milestones in the visualization of *Sulfolobus acidocaldarius*. (**A**) First transmission electron micrograph of *S. acidocaldarius* showing the characteristic irregular lobed cell morphology (22). (**B**) Freeze-etch electron microscopy revealing the highly ordered S-layer lattice (26). (**C**) Scanning electron micrograph of a 3-day-old *S. acidocaldarius* biofilm (27). (**D**) Fluorescence *in situ* hybridization demonstrating species-specific cellular aggregation upon UV treatment (*S. acidocaldarius* in green and *S. tokodaii* in red) (28). (**E**) Live-cell thermomicroscopy visualizing rapid Aap-pilus retraction dynamics at growth temperature (29). (**F**) Negative-stain electron microscopy revealing surface appendages involved in motility, adhesion, and cell–cell interactions. (**G**) Immunolocalization of cell division protein Vps4 in dividing cells (30). (**H**) SRRF super-resolution image of the ESCRT-III homolog CdvB1 (31). (**I**) Time-lapse microscopy of cells expressing cell division protein CdvA fused to thermostable green fluorescent protein Matcha (32).

Members of the Thermoproteota are typically isolated from high-temperature, acidic environments, such as volcanic hot springs. Among these, the order Sulfolobales is especially prominent. The first representative of this group to be cultivated was *Sulfolobus acidocaldarius*, isolated in 1972 by Thomas Brock from a muddy hot spring in the Norris Geyser Basin, Yellowstone National Park (22) (Fig. 1A). This thermoacidophile, growing optimally at ~75°C and pH 2–3, has remained a cornerstone of archaeal research ever since. In 1980, Wolfram Zillig isolated *Sulfolobus solfataricus* (now *Saccharolobus solfataricus*) from Solfatara near Naples (4). He later also isolated *Sulfolobus islandicus* (now *Saccharolobus islandicus*) from solfataric hot springs in Iceland (23), a species that has since become the second major model organism within the Sulfolobales next to *S. acidocaldarius*. In addition, *Sulfolobus tokodaii* (now *Sulfurisphaera tokodaii*) was obtained from hot springs in Japan (24, 25), as well as a few less widely used Sulfolobales species. The wide geographic distribution of the different Sulfolobales species across volcanic environments worldwide, coupled with their close phylogenetic relatedness, suggests long-term evolutionary and ecological stability within this lineage despite continental separation.

During the early years of thermoacidophile research, taxonomy was challenging due to the absence of genomic sequences. As a result, several European isolates of similar organisms were described independently under different names, such as *Caldariella acidophila*, proposed for Italian isolates from Solfatara near Naples. Subsequent comparative studies of physiology, DNA composition, and RNA polymerase structure demonstrated that these strains belonged within the genus Sulfolobus (4, 33). Over the years, ongoing phylogenetic refinement has led to multiple taxonomic revisions within the Archaea. The phylum formerly known as Crenarchaeota is now classified as Thermoproteota, and several Sulfolobales species have been reassigned, for example, *S. solfataricus* and *Sa. islandicus* to *Saccharolobus*, and *Sulfolobus tokodaii* to *Sulfurisphaera*. Conveniently, *S. acidocaldarius*, being the first isolated Sulfolobus species, has retained its original name (34–36).

Comparative genomic analyses revealed striking differences in genome plasticity among Sulfolobales species. The almost 3 Mbp genome of *Sa. solfataricus* contains more than 200 insertion sequences (ISs) and repeated elements, many of which are actively mobile (37, 38). Consistent with this, more than 90% of spontaneous mutations reported in *Sa. solfataricus* arise through IS transposition, making mobile elements the main source of genome variation. *Sa. islandicus* genomes also harbor numerous mobile genetic elements and variable genomic islands enriched in insertion sequences, CRISPR loci, and toxin-antitoxin systems. Population genomic studies have further revealed extensive recombination and strong geographic structuring *of Sa. islandicus* populations, indicating high levels of genome plasticity in this species (39–41). However, unlike *Sa. solfataricus,* where IS transposition dominates spontaneous mutagenesis, in *Sa. islandicus*, IS transposition contributes only approximately 5%–10% of spontaneous mutations, whereas small insertions and deletions constitute approximately half of all mutations and are largely attributable to the activity of the error-prone DNA polymerase Dpo2 (42). In contrast*, S. tokodaii* (2.69 Mbp) contains very few insertion sequences, and *S. acidocaldarius* (2.32 Mbp) appears to lack active IS elements entirely (43). This relative genomic stability likely contributed to the later success of precise genetic manipulation *in S. acidocaldarius*. Consequently, *Sa. islandicus* has become an important model for studying archaeal evolution and population genomics, complementing *S. acidocaldarius* as a genetically stable laboratory model.

## EARLY STUDIES ON *S. ACIDOCALDARIUS*

Early research on *S. acidocaldarius* was driven by its remarkable ability to thrive under extreme conditions of high temperature and low pH. Its thermostability and acid tolerance soon attracted attention in both fundamental and applied research. In particular, the robustness of enzymes from *S. acidocaldarius* under harsh conditions positions it as a useful source for industrial processes. Early biochemical studies characterized highly stable RNA polymerases, proteases, and metabolic enzymes, often with a clear perspective toward biotechnological exploitation (44–46).

In addition to enzymes, the unique membrane lipids of *S. acidocaldarius*, particularly their membrane-spanning tetraether lipids, became a central topic of investigation because of their remarkable membrane stability at high temperatures and low pH (47). Recently, the identification of key enzymes involved in tetraether lipid biosynthesis and cyclization has provided important insights into the mechanisms involved in archaeal membrane architecture and lipid diversification (48–50). At the same time, metabolic pathway analyses revealed adaptations in central carbon metabolism, sulfur metabolism, and systems that enable life at the thermoacidophilic limit (51–54). Together, these studies established Sulfolobales not only as model organisms for understanding archaeal cell biology, extremophile adaptation, and evolutionary processes, but also as valuable sources of robust biomolecules in industrial and biotechnological applications.

## DEVELOPMENT OF GENETIC SYSTEMS AMONG SULFOLOBALES

Early efforts to develop a genetic system for Sulfolobales began in the 1990s, using the conjugative plasmid pNOB8, which could autonomously spread from cell to cell (55). While this plasmid provided the first entry point into DNA transfer in *Saccharolobus solfataricus*, allowing the expression of a functional β-galactosidase, its large size (~45 kb) made cloning difficult. Moreover, technical challenges such as the need for thermostable selection markers had to be overcome. Most selection markers that worked in euryarchaeal systems failed in Sulfolobales, either due to physiological differences or to instability at high temperatures or acidic pH. As a result, systems based on auxotrophic markers were developed (56–59). The first targeted deletion in *Sa. solfataricus* was published in 2003, using a strain (PBL2025) that lacked a genomic region required for lactose utilization. Selection for growth on lactose enabled the introduction of deletions (60).

Around the same time, an auxotrophic *Sa. solfataricus* mutant deficient in uracil biosynthesis (PH1–16) was established (37). Initially used in virus research, this strain became the host for pMJ03, a shuttle vector based on the SSV1 virus, enabling homologous and heterologous expression in Sulfolobales (61). pMJ03 and its derivatives have since been used in a variety of studies (62–67). However, the 33 kb size of the viral vector again complicated cloning procedures. To address this, the Lipps lab developed a vector system based on the small plasmid pRN1, isolated from a *Sa. islandicus* strain. Although it offered advantages in terms of size and stability, the uracil auxotrophy in *Sa. solfataricus* was not very stringent, making selection less reliable (23, 56, 68). Similarly, the related plasmid pRN2, isolated from the same host, was used by another lab to establish a vector system in *Sa. islandicus* (69).

The complete genome sequencing of *S. acidocaldarius* in 2005 opened the door to more systematic genetic work (70). Building on Dennis Grogan’s pioneering studies of the *pyrEF* locus in *S. acidocaldarius* (71–73), which made use of uracil auxotrophy and demonstrated targeted DNA integration using PCR-generated constructs with short homologous flanking regions, a uracil-auxotrophic deletion mutant of strain DSM 639, named MW001, was constructed in 2012 by Michaela Wagner in our lab (58). MW001 became the foundation for a robust and versatile genetic toolbox in *S. acidocaldarius* (58, 74, 75).

Today, MW001 is widely distributed across archaeal research labs as a standard genetic background strain. It has been used in around 200 published studies, in which more than 100 unique single-deletion mutants were created (File S1). In addition, we created a diverse collection of plasmids for protein expression using various tags and promoters (76). It serves as a major model system for archaeal molecular biology, enabling genetic dissection of cell division, surface structure assembly, metabolism, biofilm formation, N-glycosylation, transcription, translation, lipid biosynthesis, and stress responses (7). Similar *S. acidocaldarius pyrEF* mutant background strains were constructed in other laboratories, including MR31 and SK-1 (77, 78) (Table 1). As a result, the number of publications involving *Sulfolobus* deletion strains has grown exponentially over the past decade. We have compiled a comprehensive list of all derived single-gene deletion strains in *S. acidocaldarius*, provided in File S1.

**TABLE 1:** Genetically tractable Sulfolobales strains and derivatives commonly used for genetic manipulation

Strain/derivative	Species	Genotype	Auxotrophy/selectable marker	Note	Primary reference
MR31	*S. acidocaldarius* DSM639	Δ*pyrE* (18 bp deletion)	Uracil auxotrophy/5-FOA counterselection	Early genetics background	(77)
MW001	*S. acidocaldarius* DSM639	Δ*pyrE*, (322 bp deletion)	Uracil auxotrophy/5-FOA counterselection	Default *S. acidocaldarius* genetics background	(58)
MW2000	*S. acidocaldarius* DSM639	*pyrE* mutant	Uracil auxotrophy/5-FOA counterselection	Novel optimized genetics background used in Albers lab	N. van der Kolk and S. -V. Albers, unpublished data
JDS22	*S. acidocaldarius* DSM639	Δ*pyrE* (16–38 nt)	Uracil auxotrophy/5-FOA counterselection	Used for DNA exchange assays compatible with MW001	(73)
SK-1	*S. acidocaldarius* DSM639	Δ*pyrE* (18 bp deletion); Δ*suaI*	Uracil auxotrophy/5-FOA counterselection	No plasmid methylation required	(78)
PH1-16	*Sa. solfataricus* P1	*pyrF/lacS* disrupted by insertion sequences	Uracil auxotrophy and lactose selection	Used for virus expression vectors	(37)
PBL2025	*Sa. solfataricus* 98/2	*lacS* deletion; SSO3004–SSO3050 deleted	β-Galactosidase, LacS for lactose selection	Used for virus-based expression; no counterselection, no efficient growth	(79)
E233S	*Sa. islandicus* REY15A	Δ*pyrEF* and Δ*lacS*	Uracil auxotrophy/5-FOA counterselection, lacS, simvastatin selection	Default S. islandicus genetics background	(69, 80)
RJW004	*Sa. islandicus* M16.4	Δ*pyrEF*Δ*lacS*Δ*argD*	Uracil and agmatine auxotrophy; *lacS* for blue-white screening	Multi-marker genetics and new reporter/selection tools	(81)

Parallel systems have been developed in other Sulfolobales species (Table 1). *Sa. islandicus* REY15A E233S, for instance, has been widely used in combination with its endogenous CRISPR-Cas systems for genome editing, gene silencing, transposon mutagenesis, and reporter assays (69, 82). In addition to classical *pyrEF* selection, the genetic toolbox of *Sa. islandicus* was further expanded through the introduction of selectable markers based on simvastatin resistance (*hmgA*) and the stringent agmatine prototrophy marker (*argD*) (81, 83); however, we have so far been unable to establish similar prototrophic selection systems in S. *acidocaldarius*. A genome-wide transposon mutagenesis study in *Sa. islandicus* by the lab of Rachel Whitaker has given the first comprehensive blueprint of essential functions in an archaeon, highlighting essential genes with unknown functions and showing that the S-layer is not essential under laboratory conditions (84, 85). Together, *S. acidocaldarius and Sa. islandicus* have served as the cornerstone of Sulfolobales research, establishing the basis for much of our understanding of archaeal (particularly Thermoproteota) molecular and cellular biology.

## THE CELL SURFACE AND SURFACE STRUCTURES OF *S. ACIDOCALDARIUS*

**Fig 2:**
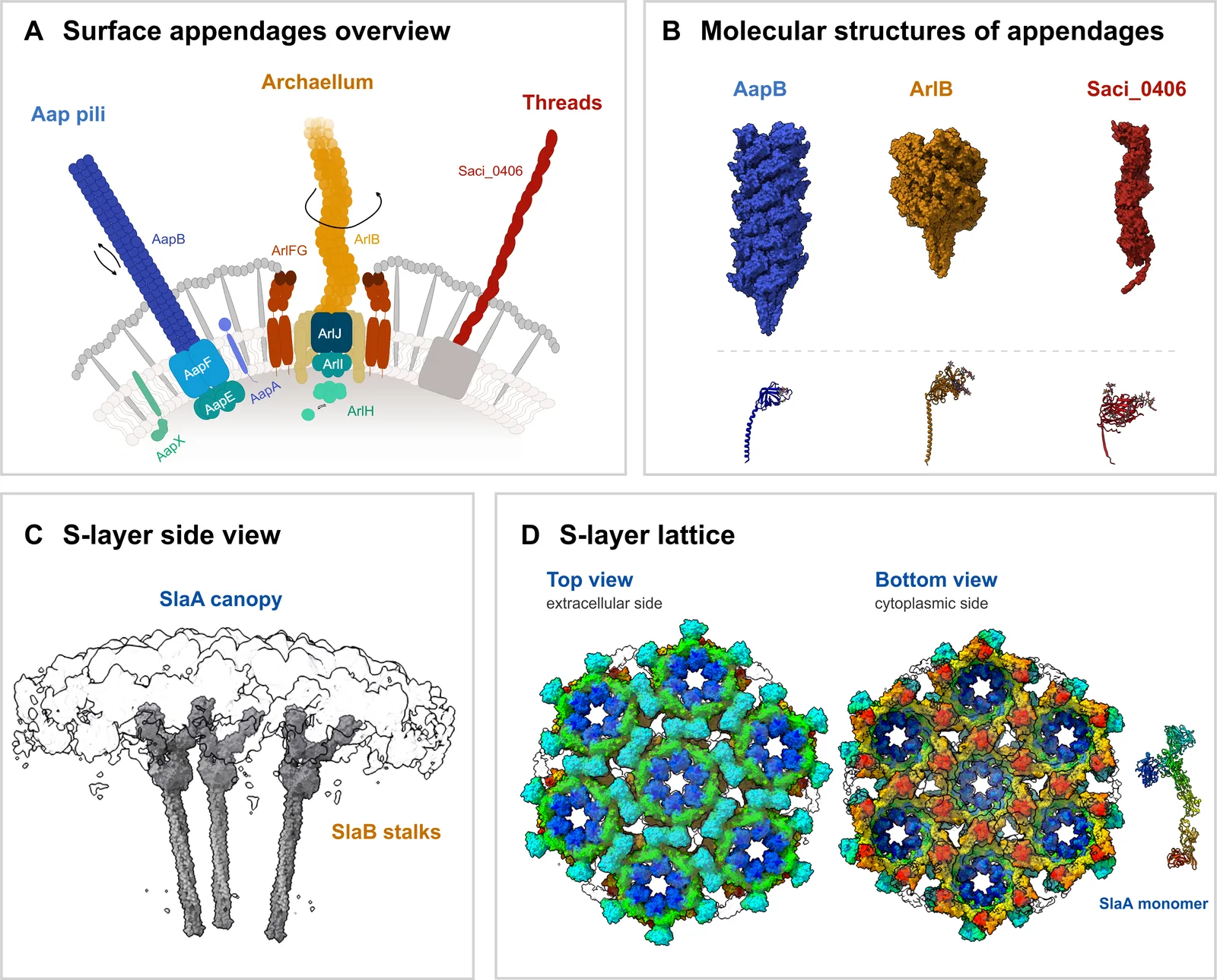
Molecular architecture of the *Sulfolobus acidocaldarius* cell surface. (**A**) Overview of the three major surface appendages of *S. acidocaldarius*: Aap pili, archaella, and thread filaments; and their assembly machineries. (**B**) Cryo-EM structures of the corresponding filament-forming proteins AapB, ArlB, and Saci_0406 (89–91). (**C**) Side view of the archaeal S-layer, composed of a membrane-anchored SlaB stalk and an outer SlaA canopy. (**D**) Top and bottom views of the S-layer lattice revealing its hexagonal organization (86).

The very first electron micrographs of *S. acidocaldarius* from 1974 already revealed the characteristic hexagonal array of subunits in its cell envelope, which has since become a hallmark of the Sulfolobales (26) (Fig. 1B). We now know that the cell envelope of *S. acidocaldarius* consists of a single S-layer composed of two major proteins: the membrane-anchored SlaB and the surface-exposed SlaA, which together form the canopy-like outer lattice (Fig. 2C). Structural analyses later revealed that the S-layer is organized into trimers of SlaA, forming a tightly packed lattice (Fig. 2D) (86). The S-layer is essential for anchoring the archaellar motor machinery to the cell envelope through the stator complex (87). Interestingly, despite these important structural functions, the S-layer of Sulfolobales is not essential under standard laboratory conditions (85, 88). However, recent work has shown that it plays a critical role in maintaining cell integrity and contributes directly to cytokinesis by flattening the division furrow, thereby supporting efficient ESCRT-III-mediated membrane constriction (88).

Dennis Grogan was among the first to recognize that these S-layer proteins are highly glycosylated and developed methods to isolate membrane ghosts from *S. acidocaldarius* cells (92). The S-layer proteins, as well as essentially every other secreted protein, are N-glycosylated in *S. acidocaldarius* (93). The full *N*-glycan consists of six sugars, comprising a chitobiose core as in eukaryotic N-glycans, from which a sulfated sugar, 6-sulfoquinovose, and a glucose moiety are branching, as well as two mannose residues (93, 94). Studies, largely carried out by Benjamin Meyer from our lab and the lab of Jerry Eichler, have unraveled parts of the N-glycosylation pathway (95–100); however, in contrast to the studies in *H. volcanii* and *M. maripaludis,* AlgB, the oligosaccharyltransferase, was shown to be essential in *S. acidocaldarius*. Moreover, N-glycan truncation analyses indicated that the glycan could not be reduced below a trisaccharide, suggesting a minimal glycan requirement for viability and/or cell envelope integrity (95, 96, 98). The latter might be because glycosylation of surface proteins is essential for their stability under thermoacidophilic growth conditions.

*S. acidocaldarius* has also served as a model for understanding the assembly and function of diverse archaeal cell surface structures (Fig. 1F and 2A). These include, most importantly, the archaellum (the archaeal motility structure) (101), the Aap pili (adhesive archaeal pili) (29, 89, 102), and the Ups pili (UV-inducible pili) (28, 103, 104). Although these systems have distinct biological roles: swimming motility, adhesion and twitching motility, and cell-cell interactions, respectively, they are all assembled by a conserved type IV pili (T4P) assembly machinery.

Comparative genomic analyses revealed that archaea have undergone an expansion of T4P systems, demonstrating extensive diversification, with *S. acidocaldarius* being one of the best experimentally characterized representatives of this broader diversity (105, 106).

The archaellum has been a major research focus in our lab over the last 2 decades, making use of our versatile genetic toolbox. It is the motility structure of archaea and is assembled via a mechanism similar to that of the bacterial T4P, while specialized accessory proteins enable filament rotation and swimming motility (101, 107, 108). Initial genetic analyses of the archaellum gene cluster in *S. acidocaldarius* demonstrated that all seven genes within the cluster are required for proper archaellum assembly and rotation (109). Subsequent structural and functional studies of the seven encoded subunits provided important insights into their individual roles in filament biogenesis, motor assembly, and torque generation (87, 110–112) (Fig. 2C and D).

In *S. acidocaldarius*, archaellum production is typically induced during the stationary phase and can be strongly stimulated under nutrient starvation conditions. This regulatory pattern differs from that observed in *Haloferax volcanii*, where archaellum expression occurs primarily during the very early logarithmic growth phase (113). The complex regulatory network controlling *S. acidocaldarius* archaellum expression has been extensively studied. It involves Arn (archaellum regulatory network) activators and repressors, phosphorylation-dependent signaling, and links to nutrient sensing and stress adaptation (65, 114–117). Together, these studies established *S. acidocaldarius* as one of the best-characterized model organisms for understanding the role, structure, assembly, and regulation of archaeal motility structures.

Next to archaella, *Sulfolobus* cells produce another type IV pilus system, the Aap pili (Fig. 1E and 2A). The Aap pili appear to be inversely regulated relative to the archaellum, being predominantly expressed during exponential growth, when cells are actively dividing. They were initially thought to function mainly in surface attachment, cell-cell contacts, and biofilm formation (Fig. 1C) (102, 118). More recently, however, the Aap pilus was shown to undergo active retraction despite the absence of a dedicated retraction ATPase and to use this retraction for twitching motility (Fig. 1E) (29, 89). This behavior, also observed in bacteria but so far unique among archaea, has been important for our understanding of type IV pilus evolution and is consistent with the hypothesis that T4P retraction predates the emergence of dedicated bacterial retraction ATPases (119).

A third T4P of major importance among Sulfolobales is the UV-inducible Ups pilus. In contrast to archaella and Aap pili, Ups pili are specifically produced in response to DNA damage, particularly after UV irradiation. They mediate species-specific cellular aggregation and promote DNA exchange between neighboring cells via the Ced system, thereby facilitating homologous recombination and genome repair (Fig. 1D) (28, 103, 104, 120, 121). This unique community-based DNA repair system established a direct link between type IV pili, stress responses, and the maintenance of genome integrity in archaea.

All three surface structures also play vital, though different, roles in biofilm production under static growth conditions (118). Deletion mutants lacking any one of these structures display defects such as reduced surface attachment, impaired microcolony formation, and altered dispersal behavior (27, 118, 122). In addition to T4P, the extracellular polymeric substance (EPS) matrix is a major determinant of biofilm stability and architecture. Work by the Siebers lab demonstrated that *S. acidocaldarius* biofilms produce a complex EPS matrix containing carbohydrates, proteins, extracellular DNA, and other biomolecules that provide structural cohesion and likely contribute to protection from environmental stress (123, 124). Several studies have given insights into the complex and multilayered regulatory network regulating biofilm formation, involving transcriptional regulators, post-translational signaling pathways, toxin-antitoxin systems, and environmental sensing systems that are still far from fully understood (122, 125–127).

A final non-T4P surface structure of *S. acidocaldarius* is the very thin, approximately 5 nm wide, so-called thread (90). These filaments were already observed in early electron micrographs of Sulfolobus cells, but their molecular architecture and protein composition were only resolved recently. The mechanism by which these filaments are assembled and anchored to the cell envelope remains unknown. Their widespread presence suggests they may represent an additional, previously overlooked class of archaeal surface appendages (90).

## MOLECULAR BIOLOGY OF *S. ACIDOCALDARIUS*

Over the past 2 decades*, S. acidocaldarius* has emerged as a powerful model for archaeal molecular biology, providing key insights into core cellular processes, some of which resemble simplified versions of eukaryotic systems. Its thermostable proteins have been highly useful for biochemical and structural studies, and major advances have been made in our understanding of chromosome organization, DNA replication, DNA repair, DNA segregation, transcription, translation, and cell division.

### Chromosome organization

The highly abundant nucleoid-associated protein Sac7d of *S. acidocaldarius* and related chromatin proteins such as Sul7d, Alba, and Cren7 in other Sulfolobus species were among the first *Sulfolobus* proteins to be characterized (128, 129), revealing a unique archaeal strategy for DNA compaction distinct from bacterial nucleoid proteins and from histone-based chromatin seen in many other archaea (130). It is now clear, however, that chromosome organization in Sulfolobales is hierarchical and extends far beyond local DNA bending and bridging by these abundant proteins. Work from the Bell lab showed that chromosomes are separated into transcriptionally active A compartments, enriched in essential genes and replication origins, and less active B compartments, which display lower transcriptional output. Central to maintaining this higher-order architecture is the *Sulfolobus*-specific SMC-family ATPase Coalescin (ClsN), which preferentially associates with B-compartment regions and is required for compartmentalization (131). At a finer scale, additional DNA-binding proteins, including members of the Lrs14 family, are thought to remodel chromosome architecture locally, thereby promoting adaptive transcriptional responses (132).

### DNA replication

The study of DNA replication in Sulfolobales has been highly important for understanding the evolutionary origins of eukaryotic DNA replication. The establishment of cell cycle synchronization methods in the early 2000s, including acetate treatment and the “baby machine,” has enabled detailed analyses of replication dynamics (133, 134). In *S. acidocaldarius*, which has a typical doubling time of about 4 hours under optimal growth conditions, we now know that replication is integrated within a highly ordered cell cycle that coordinates DNA replication, chromosome segregation, and cell division. This cycle contains a short pre-replicative G1 phase, a chromosome-replication S phase, an extensive G2 phase, followed by brief genome segregation and cell division stages (135, 136). Unlike bacteria, the circular chromosome of Sulfolobales is replicated from three origins that fire once per cell cycle and are individually non-essential, each controlled by Orc1/Cdc6-related initiator proteins that recruit the MCM helicase to assemble bidirectional replication forks (133, 134, 137). Biochemical and structural studies in Sulfolobus have, over the past 2 decades, defined a simplified eukaryote-like replisome comprising MCM, GINS, Cdc45 homologs, PCNA, primase, and DNA polymerases, providing a conserved but simplified version of the more complex eukaryotic machinery (138). Similar to eukaryotes, replication fork progression and origin activation in Sulfolobales are likely tightly coordinated with cell-cycle progression and chromosome segregation (139), a process that is at least partially mediated by the recently identified aCcr family of cell-cycle-regulated transcription factors (140–142).

### DNA maintenance and repair

Early genetic and physiological studies by Dennis Grogan’s lab established *S. acidocaldarius* as a model for investigating genome stability and DNA repair under extreme conditions (143, 144). Subsequent biochemical and structural work, primarily from the lab of Malcolm White, elucidated the functions of conserved archaeal repair proteins, including the Mre11-Rad50 complex and the associated helicase-nuclease pair HerA and NurA, which play central roles in the processing and repair of DNA double-strand breaks (145, 146). Sulfolobales also encode homologs of the eukaryotic nucleotide excision repair proteins XPB, XPD, and XPF, highlighting the evolutionary relationship between archaeal and eukaryotic DNA repair systems (147).

Unique to the Sulfolobales is the above-mentioned community-based DNA damage response, in which species-specific cellular aggregation mediated by the Ups pili and chromosomal DNA exchange via the Ced system promote homologous recombination and genome restoration upon DNA damage (103, 121). Building on these observations, studies from the labs of Qunxin She, Yulong Shen, and others uncovered a Sulfolobales-specific regulatory network centered on the transcriptional regulators Orc1-2 and TFB3, linking DNA damage sensing to the induction of repair pathways, cellular aggregation, and DNA exchange (148–152).

### Chromosome segregation

Chromosome segregation in Sulfolobales takes place after completion of DNA replication at the end of the extended G2 phase (153, 154). This process is mediated by the SegAB system, composed of the ParA-like ATPase SegA and the DNA-binding protein SegB, which associate with centromere-like sequences (155, 156). In the past year, several groups have made significant progress using various Sulfolobales, including *S. acidocaldarius*, to better understand this system and its coordination with the cell cycle. Live thermomicroscopy revealed a specific order in which chromosomal DNA transitions from a membrane-associated state to a mobile phase, then compacts into two segregated chromosomes that define the future division axis. Importantly, this process is coordinated with cytokinesis through a checkpoint-like mechanism that ensures segregation proceeds only after assembly of the division ring, thereby functionally linking nucleoid separation to cell division (157). Consistent with this, Seg proteins were found to interact directly with CdvA, and deletion of the Seg system leads to aberrant nucleoid morphology and defects in division site placement, including premature assembly of the cell division recruiter CdvA (158). The Seg system functions differently from the well-known bacterial ParABS system, in which ParB binds centromere-like *parS* sites and ParA forms a dynamic ATP-dependent gradient that drives segregation of ParB-DNA complexes. In contrast, SegB marks chromosomal loci, and SegA reorganizes into elongated structures spanning the two nucleoids, possibly providing the force or spatial signals required for their separation (159, 160). Taken together, these recent studies indicate that chromosome segregation in Sulfolobales is closely linked to changes in chromosome organization and is tightly coordinated with cell division.

### Cell division

After discovering that ESCRT-III proteins are present in the vesicles of different Sulfolobales (161), the Bell and Bernander groups played central roles in establishing the importance of the ESCRT-III-based Cdv machinery for cell division in Sulfolobales. Its homology to the eukaryotic membrane-remodeling machinery represented a breakthrough in understanding archaeal cytokinesis (30, 162). The Cdv machinery consists of CdvA, an archaeal-specific protein involved in membrane recruitment and positioning of the division apparatus; CdvB, an ESCRT-III homolog that forms the initial constriction scaffold; and CdvC, a homolog of the eukaryotic Vps4 ATPase that remodels and disassembles the complex. Since then, *S. acidocaldarius* has become one of the principal model organisms for dissecting archaeal cytokinesis. Subsequent studies have described two additional ESCRT-III homologs, CdvB1 and CdvB2, which are recruited after CdvB ring assembly and play key roles during membrane constriction and completion of cell division (31, 153, 163). The Baum lab also showed that cytokinesis in Sulfolobus is regulated, as in eukaryotic cell division, by the proteasome through targeted degradation of CdvB (31, 164). These findings revealed that selective proteolysis of CdvB is a key trigger for the transition from division-ring assembly to active constriction, thereby linking archaeal cytokinesis to cell-cycle control mechanisms previously thought to be characteristic of eukaryotes (31, 164).

Initially, all localization studies of cell division proteins in Sulfolobus relied on immunolocalization (Fig. 1G H), as no thermostable fluorescent proteins were available for live-cell imaging under the organisms’ extreme growth conditions. Recent advances in the field, including the development of high-temperature microscopy platforms as well as thermostable fluorescent proteins such as TGP, its improved derivative Matcha, and the thermostable rhodamine-binding tag Rhobin, now promise a new era of live-cell imaging in Sulfolobales (Fig. 1I) (32, 165, 166).

## FINAL AND FUTURE

More than 5 decades after Thomas Brock first isolated *Sulfolobus acidocaldarius* from the hot springs of Yellowstone National Park, and nearly 50 years after the formal recognition of the Archaea, this organism has emerged as one of the most powerful and versatile model systems in archaeal biology. Over this period, hundreds of papers have been published on many aspects of its physiology, genetics, biochemistry, and cell biology, including studies of surface structures, motility, biofilm formation, chromosome organization, DNA replication and repair, transcription, translation, metabolism, stress responses, and cell division. While this review has highlighted some of the most influential discoveries made using *S. acidocaldarius*, many important contributions could be described only briefly or not at all. For a more in-depth overview of Sulfolobales cellular processes and molecular biology, we refer to (7) and (167).

The continued success of *S. acidocaldarius* as a model system lies in its unique combination of genetic accessibility, physiological robustness, and evolutionary relevance. Its stable genome, well-developed genetic toolbox, and biochemical tractability make it an ideal organism for dissecting archaeal-specific processes, many of which resemble simplified versions of eukaryotic mechanisms. It therefore plays a central role in efforts to understand the evolution of complex cellular systems.

Looking ahead, the field is entering an especially exciting phase. Developments in high-temperature live-cell imaging, thermostable fluorescent reporters, cryo-electron microscopy, increasingly refined genetic systems, and AI-driven protein structure and function are enabling the study of archaeal cells with significantly improved spatial and temporal resolution. These will thereby provide deeper insights into fundamental archaeal biology and accelerate the discovery of thermostable enzymes, molecular complexes, and cellular processes with potential biotechnological relevance. The discovery and ongoing characterization of Asgard archaea have reshaped our understanding of eukaryotic origins. They encode an expanded repertoire of eukaryotic signature proteins but remain genetically intractable so far. In this context, genetically accessible model systems such as *S. acidocaldarius* and other members of the Thermoproteota will serve as crucial experimental proxies. Comparative analyses of Sulfolobales and Asgard lineages, particularly regarding membrane remodeling, cytoskeletal organization, and protein trafficking, might enable us to experimentally test hypotheses about the origin and evolution of eukaryotic cellular complexity.

## SUPPLEMENTAL MATERIAL

File S1 jb.00322-26-s0001.xlsx Overview of Sulfolobus acidocaldarius gene deletion mutants and associated phenotypes reported in literature.

ASM does not own the copyrights to Supplemental Material that may be linked to, or accessed through, an article. The authors have granted ASM a non-exclusive, world-wide license to publish the Supplemental Material files. Please contact the corresponding author directly for reuse.
